# β-Amyloid, Tau, Neurodegeneration Classification and Eligibility for Anti-amyloid Treatment in a Memory Clinic Population

**DOI:** 10.1212/WNL.0000000000201043

**Published:** 2022-11-08

**Authors:** Anna Rosenberg, Ulf Öhlund-Wistbacka, Anette Hall, Alexandre Bonnard, Göran Hagman, Marie Rydén, Charlotta Thunborg, Fleur Wiggenraad, Anna Sandebring-Matton, Alina Solomon, Miia Kivipelto

**Affiliations:** From the Division of Clinical Geriatrics (A.R., U.Ö.-W., C.T., A.S.-M., A.S., M.K.), Centre for Alzheimer Research, Department of Neurobiology, Care Sciences and Society, Karolinska Institutet, Stockholm, Sweden; Department of Neurology (A.R., A.H., A.S, M.K.), Institute of Clinical Medicine, University of Eastern Finland, Kuopio; Medical Unit Aging (U.Ö.-W., A.B., G.H., M.R., F.W., M.K.), Karolinska University Hospital, Stockholm, Sweden; Division of Neurogeriatrics (A.S.M.), Centre for Alzheimer Research, Department of Neurobiology, Care Sciences and Society, Karolinska Institutet, Stockholm, Sweden; Ageing Epidemiology Research Unit, School of Public Health, Imperial College London, United Kingdom.

## Abstract

**Background and Objectives:**

ATN (β-amyloid [Aβ], tau, neurodegeneration) system categorizes individuals based on their core Alzheimer disease (AD) biomarkers. An important potential future use for ATN is therapeutic decision-making in clinical practice once disease-modifying treatments (e.g., anti-amyloid), become widely available. In this cross-sectional study, we applied ATN and estimated potential eligibility for anti-amyloid treatment in a real-life memory clinic with biomarker assessments integrated into the routine diagnostic procedure and all specialized resources available for the implementation of novel treatments.

**Methods:**

We included all consecutive patients at the Karolinska University Hospital Memory clinic in Solna, Stockholm, Sweden, who had their first diagnostic visit in April 2018–February 2021, informed consent for the clinic research database, and available clinical and biomarker (CSF and imaging) data. ATN classification was based on CSF Aβ42 (or Aβ42/40; A), CSF phosphorylated tau (T), and medial temporal lobe atrophy (N). For CSF markers, we applied laboratory cutoffs and data-driven cutoffs for comparison (determined with Gaussian mixture modeling). Eligibility for anti-amyloid treatment was assessed following the published recommendations for aducanumab (AD dementia or mild cognitive impairment [MCI] with no evidence of non-AD etiology, appropriate level of cognition, and AD-consistent CSF profile).

**Results:**

The study population consisted of 410 patients (52% subjective cognitive impairment, 23% MCI, and 25% any dementia; age 59 ± 7 years, 56% women). Regardless of biomarker cutoffs, most patients were A−T−N− (54%–57%). A+ prevalence was 17%–30% (higher with data-driven cutoffs). Up to 13% of all patients (27% of those with MCI and 28% of those with dementia) were potentially eligible for anti-amyloid treatment when AD-consistent CSF was defined as any A+ profile. When A+T+ profile was required, treatment was targeted more to the dementia than MCI stage (eligibility up to 14% in MCI and 22% in dementia). The opposite applied to earlier-stage intervention (A+T−N−; eligibility up to 12% in MCI and 2% in dementia).

**Discussion:**

In a memory clinic setting with all necessary infrastructure and national guidelines in place for dementia diagnostic examination (best-case scenario), most of the patients did not meet the eligibility criteria for anti-amyloid treatment. Continuing the development of disease-modifying treatments with different mechanisms of action is a priority.

The ATN (β-amyloid [Aβ], tau, neurodegeneration) system categorizes individuals based on the core Alzheimer disease (AD) fluid/imaging biomarkers, independently of cognition or clinical staging.^1,2^ Different combinations of normal (−) and abnormal (+) biomarkers result in 8 profiles under 3 main categories: normal (A−T−N−), non-AD pathologic change (tau or neurodegeneration in the absence of Aβ; A−T+N± or A−T−N+), and Alzheimer continuum (Aβ alone or together with other pathologies; A+T±N±). A combination of abnormal Aβ and tau (A+T+, regardless of N) denotes AD. ATN was designed to help define biologically homogenous groups for clinical trials to ensure target engagement and the detection of treatment effects. However, given the recent FDA approval of the first disease-modifying drug^3^ (Aβ antibody aducanumab; marketing authorization refused in Europe^4^) and ongoing regulatory assessment of other similar drugs,^5^ ATN might soon be applied also in clinical practice to confirm diagnosis and guide therapeutic decision-making. ATN profiles have been characterized cross-sectionally^6-9^ and longitudinally^10-20^ to validate the classification system but mainly in selected research cohorts.

Whether ATN is a suitable approach for diagnostic and therapeutic decision-making in clinical practice is a crucial and currently unanswered question. The aim of this study was to apply ATN to a real-life memory clinic cohort and estimate potential eligibility for anti-Aβ disease-modifying therapy. Furthermore, we explored different ATN operationalizations and their effect on biomarker classification and treatment eligibility (established vs data-driven biomarker cutoffs; CSF Aβ42 vs Aβ42/40 as a marker of A). Treatment eligibility was based on the practical appropriate use recommendations published after the aducanumab approval,^21^ assuming that similar eligibility criteria would likely apply to any drug with this mechanism of action.

## Methods

### Standard Protocol Approvals, Registrations, and Patient Consents

The Karolinska University Hospital electronic database and biobank for clinical research (GEDOC) and this study have received ethical approval (Regional Ethical Review Board in Sweden; Dnr 2011/1987-31/4 and 2020-06484). All patients provided written informed consent.

### Participants and Study Design

The study was conducted at the Karolinska University Hospital Medical Unit Aging Memory clinic in Solna, Stockholm, Sweden. Since April 2018, this specialized outpatient clinic examines individuals with cognitive complaints referred by general practitioners in primary and occupational health care in the catchment area (northern Stockholm), and additionally individuals younger than 70 years in the entire Stockholm region. All diagnostic examinations are performed within 1 week (fast track model).

The dementia examination process follows national guidelines established by the Swedish Board of Health and Welfare.^22^ Before referral, patients undergo a basic medical evaluation, including medical history, MRI/CT, and Mini-Mental State Examination (MMSE). If patients have any major medical condition (e.g., psychiatric, cardiovascular or cerebrovascular disease, depression, or cancer), referrals are accepted only after the referring clinic has ensured that the condition and related treatment have been stable for at least 3 months. The harmonized diagnostic evaluation at the memory clinic consists of a comprehensive medical and neurologic examination, medical and informant-based history, neuropsychological evaluation, blood chemistry, MRI, *APOE* genotyping, and CSF biomarker analysis (e.g., Aβ42, Aβ42/40, phosphorylated (p)-tau_181_, and total (t)-tau). Most patients also meet a physiotherapist for the assessment of physical functioning. Other examinations are performed as needed (e.g., PET and speech-language pathologist's consultation). All patients are routinely invited to provide written informed consent for including their clinical data and blood/CSF samples in the Karolinska University Hospital electronic database and biobank for clinical research.

A multidisciplinary team evaluates each patient and sets a consensus diagnosis based on all test results, including biomarkers. Diagnosis is based on the *Diagnostic and Statistical Manual of Mental Disorders (Fifth Edition)* *(DSM-V)* criteria, and *International Classification of Diseases, Tenth Revision (ICD-10)* coding is used. Patients who do not meet the criteria for mild cognitive impairment (MCI) or dementia are considered to experience subjective cognitive impairment (SCI). A follow-up is arranged based on clinical judgment (memory clinic/primary care).

**Figure 1:**
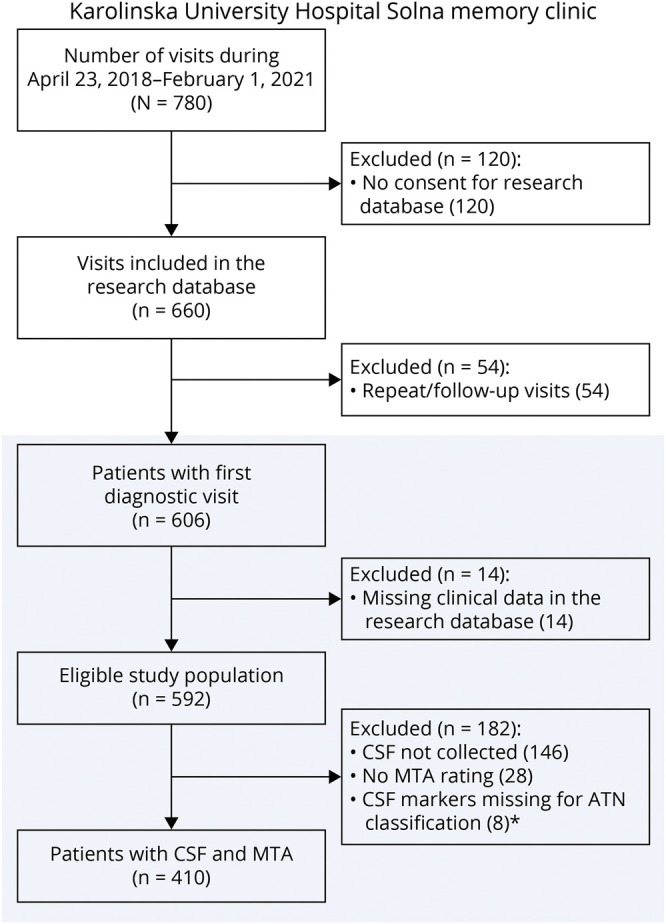
Study Flowchart and Patient Selection. * CSF markers required for our study were Aβ42, Aβ42/40, and tau markers (p-tau and t-tau). Either one of the Aβ markers was missing for 7 patients; 1 patient did not have data on any Aβ or tau markers (only neurofilament light NfL available).

For this study, we considered all consecutive patients with a first diagnostic visit between April 2018 and February 2021 and informed consent for the research database. During spring and early summer 2020, clinic activities were paused because of COVID-19, and no new patients were examined. Patient selection is shown in Figure 1. Of 606 patients with a first diagnostic visit, 592 had data in the database; a quarter of them (N = 146) did not undergo lumbar puncture (LP). Patients who did vs did not undergo LP were not significantly different regarding, e.g., demographics and cognition (eTable 1, Supplement, links.lww.com/WNL/C301). Our final study population consisted of all patients with clinical, CSF, and imaging data (N = 410).

### Cognitive Assessment

Global cognition was assessed with MMSE^23^ (before referral) and Montreal Cognitive Assessment^24^ (MoCA; at the clinic). Neuropsychological tests conducted at the clinic included the Wechsler Adult Intelligence Scale, 4th edition coding subtest to assess processing speed/attention^25^ and Rey Auditory Verbal Learning Test (learning and delayed recall)^26^ and Rey Complex Figure Test (immediate retention)^27^ to assess memory and visuospatial abilities. These routinely performed tests are available for most patients. Other cognitive domains (e.g., executive functioning), can be assessed, if necessary. In this study, we converted raw neuropsychological test scores into *z* scores using the mean and SD values of all patients with data.

### Biomarker Assessment

CSF was collected in sterile polypropylene tubes (Medicarrier art nr 67741) and analyzed at Karolinska University Hospital Laboratory in connection with the diagnostic visit. For samples analyzed until August 21, 2019, Aβ42, Aβ40, phosphorylated tau 181 (p-tau_181_), and total tau (t-tau) were measured with commercially available Innotest sandwich enzyme-linked immunosorbent assays (Fujirebio Europe). From August 22, 2019, samples were analyzed with the Lumipulse G-series (Fujirebio Europe) fully automated chemiluminescent enzyme immunoassay.^28^ Biomarker values of these assays are generally highly concordant.^29^

Patients underwent 3T MRI at the clinic (GE Medical systems Discovery MR750 3T) according to a routine protocol comprising T1 FLAIR, T1 3D GRE IR BRAVO, T2 FLAIR 3D CUBE, Ax T2 PROPELLER 4 mm, Ax DWI KS, and Ax SWI 3D KS sequences. Experienced neuroradiologists evaluated the scans and assessed visually medial temporal lobe atrophy (MTA; Scheltens scale, 0–4)^30^, global cortical atrophy (GCA; 0–3)^31^, posterior atrophy (PA; Koedam scale, 0–3)^32^, and white matter hyperintensities (WMH; Fazekas scale, 0–3).^33^ MRI or CT performed off-site according to local protocols was considered if MRI was not performed at the clinic. In addition to visual assessment, T1 and FLAIR data from the clinic scans were imported into the cNeuro cMRI software (Combinostics)^34,35^ for an automated analysis of 2regional brain volumes (cortical and subcortical) and WMH. Software generates also estimates of the common rating scales (e.g., MTA; computed scales have been validated^34^). We prioritized the automated MTA assessment (available for N = 293, 71%; N = 117 with only visual rating, MRI-based N = 90 and CT-based N = 27).

### ATN Operationalization and Biomarker Dichotomization

ATN classification was based on the recommended CSF and imaging biomarkers.^2^ We used CSF Aβ42 as A marker (Aβ42/40 for comparison) and CSF p-tau as T marker. Neurodegeneration (N) was based on MTA, following the recommendations to use different measurement modalities for ATN due to a strong correlation between CSF p-tau (T) and t-tau (N).^9^ We confirmed this correlation in our data (Spearman rho 0.85, *p* < 0.001).

For MTA, we considered a mean score of ≥1 abnormal for patients younger than 65 years and ≥1.5 for those aged 65 years and older.^36^ For CSF biomarkers, we first applied established cutoffs provided by the laboratory/manufacturer, available for Aβ42, p-tau, and t-tau. For Innotest (until 08/21/2019), cutoffs were as follows: Aβ42 ≤ 550 pg/mL, p-tau ≥60 pg/mL, and t-tau ≥400 pg/mL. For Lumipulse (after 08/21/2019), cutoffs were as follows: Aβ42 ≤ 599 pg/mL, p-tau ≥56.5 pg/mL, and t-tau ≥404 pg/mL.^29^ Second, we determined data-driven cutoffs for all biomarkers (Aβ42, Aβ42/40, p-tau, and t-tau) using Gaussian mixture modeling (GMM). These cutoffs were as follows: Aβ42 < 707 pg/mL (all samples), Aβ42/40 (x10) <0.60 (Innotest) and <0.86 (Lumipulse), p-tau ≥76 pg/mL (Innotest) and ≥56 pg/mL (Lumipulse), and t-tau ≥406 pg/mL (all samples).

Main results (ATN profile prevalence, anti-Aβ treatment eligibility) are presented for 3 ATN operationalizations (MTA as N in each version): (1) established cutoffs for CSF Aβ42 (A) and p-tau (T) based on laboratory guidelines; (2) data-driven cutoffs for CSF Aβ42 (A) and p-tau (T); and (3) data-driven cutoffs for CSF Aβ42/40 (A) and p-tau (T).

### Eligibility for Anti-Aβ Treatment

To estimate the eligibility for anti-Aβ treatment, we followed the recent appropriate use recommendations published after the FDA approval of the first anti-Aβ drug.^21^ Eligibility was assessed in all patients with available data (N = 404, missing MMSE and MoCA, N = 6). Using a stepwise approach, patients were considered eligible if they met the following criteria: (1) AD-type dementia (ICD-10 F00, G30) or MCI (ICD-10 F06.7; no evidence of non-AD neurologic disorder); (2) MMSE 21–30 (or MoCA 17–30); 3) available MRI (missing MRI indicates possible contraindications because all other routine assessments including LP were performed); 4) no anticoagulant treatment (ATC code B01; platelet antiaggregation agents B01AC were allowed^21^); and 5) CSF profile consistent with AD, i.e., any A+ or, more specifically, A+T+ (AD-type profile) or A+T−N− (earlier-stage intervention). Recommendations do not specify exclusionary medical conditions but require illnesses to be stable/managed.^21^ We did not exclude patients due to comorbidity because the standard referral process requires major illnesses (e.g., cardiovascular, psychiatric, depression, and cancer) and related treatments to be stabilized before referral. Full MRI reports were not available to assess potential imaging-related contraindications (e.g., macrohemorrhages/microhemorrhages^21^).

### Statistical Analysis

Data-driven CSF biomarker cutoffs were determined using Gaussian mixture modeling (GMM) as in previous studies.^37-39^ We calculated the models using Matlab R2019b (function fitgmdist and cluster for the probability distributions). Biomarker distributions were fitted with 2 Gaussian distributions similar to a previous ATN study,^40^ and cutoffs were defined as the points where the probability distributions changed from one Gaussian fit to another. Potential outlier values were omitted. Based on previous research,^29^ Aβ42 was assumed to have similar distributions for both assay types; for Aβ42/40 and p-tau, the assays were treated separately. Our data supported this assumption. GMM results are shown in eFigure 1 (Supplement, links.lww.com/WNL/C301).

ATN profile differences in demographics, cognition, and clinical characteristics were analyzed with the Kruskal-Wallis test and logistic regression, as applicable. Cognition differences were assessed with linear regression where the categorical ATN variable was treated as independent variable and test score as dependent variable (separate models for each test, adjusted for age, sex, and education). In case of overall statistically significant differences between ATN profiles (*p* < 0.05), *p* values for the pairwise comparisons were Bonferroni-corrected to account for multiple comparisons. Analyses were performed with Stata 14.1.

### Data Availability

Professor Kivipelto's research team is open to requests for data collected in this study. Study plan (including the research question, planned analysis, and data required) will be evaluated on a case-by-case basis. Shared data will encompass the data dictionary and de-identified data only. Analysis will be conducted in collaboration with Professor Kivipelto's team. Access is subject to the GEDOC legal framework. An access agreement will be prepared.

## Results

### Study Population Characteristics

Our study population consisted of all memory clinic patients with available clinical and biomarker data (N = 410, Figure 1). Patients were on average 59 (SD ± 7) years, had 13.5 ± 3.5 years of education, 56% were women, and 40% (159/399) were *APOE* ε4 carriers. The mean MMSE was 26.0 ± 4.2 and MoCA 22.7 ± 5.2. A total of 214 patients (52%) had SCI (age 57 ± 7 years, 61% women), 94 (23%) had MCI (age 62 ± 6 years, 53% women), and 102 (25%) had dementia (age 63 ± 6 years, 47% women). Most dementias were AD-type (N = 68); non-AD diagnoses included unspecified (N = 11), vascular (N = 7), alcohol-related (N = 6), frontotemporal (N = 5); Lewy body (N = 2); Parkinson disease (N = 1), and other dementias (N = 2).

### ATN Classification and Prevalence of ATN Profiles

Table 1 summarizes the ATN classification in the whole cohort and per diagnosis (SCI, MCI, and dementia) for each ATN operationalization. With laboratory cutoffs (Aβ42 as A), the normal A−T−N− profile was the most common (54%), followed by 2 non-AD pathologic change profiles (A−T+N− 13% and A−T−N+ 11%, Table 1). In total, 28% had a non-AD pathologic change profile and 17% had a profile defining Alzheimer continuum (any A+). AD profile (A+T+) was found in 11%. With increasing severity of diagnosis, the prevalence of A−T−N− decreased (79% in SCI, 38% in MCI, and 17% in dementia) and the prevalence of A+ and A+T+ increased (3% and 1% in SCI, 18% and 10% in MCI, 45% and 35% in dementia).

**Table 1:**
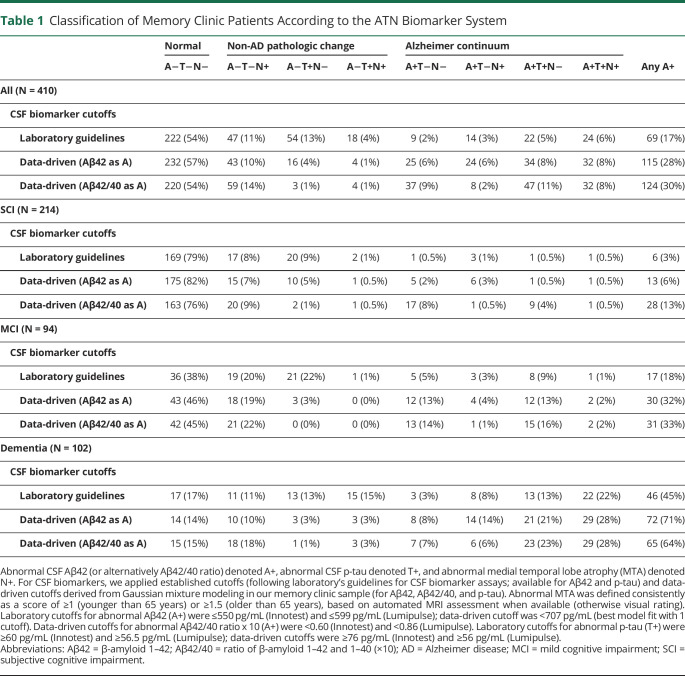
Classification of Memory Clinic Patients According to the ATN Biomarker System

Applying data-driven cutoffs instead of laboratory cutoffs (Aβ42 as A) had minor effect on the prevalence of A−T−N− (57%, Table 1). In general, redefining the cutoffs led to a decrease in the prevalence of non-AD pathologic change profiles (particularly A−T+N−) and increase in Alzheimer continuum profiles, a pattern that was evident across all diagnostic groups. Overall, 15% had a non-AD pathologic change profile, 28% had A+, and 16% had A+T+. In individual patients, most changes were from A− to A+ (N = 39) or T+ to T− (N = 26). Redefining the cutoffs had less effect on the SCI than the MCI and dementia groups. Reclassified patients became primarily A−T−N± (SCI), A−T−N− or A+T−N− (MCI), or A+T+N− or A+T+N+ (dementia).

When using data-driven Aβ42/40 instead of data-driven Aβ42 as A marker, the following reclassifications were observed: change from A−T−N− to A+T−N− (only the SCI group), from A+T−N+ to A−T−N+ (all groups), and from A−T+N− to A+T+N− (all groups) (Table 1). This led to a slight increase in the A+ prevalence overall (from 28% to 30%) and in the SCI group (from 6% to 13%). A+ prevalence remained stable in MCI (32% vs 33%) and decreased in dementia (from 71% to 64%), but this was due to the reclassification of A+T−N+. A+T+ prevalence increased from 15% to 18% in the MCI group and from 49% to 51% in the dementia group. This indicates that patients with discordant A markers, i.e., abnormal Aβ42 but normal Aβ42/40, were rarely T+ (2 of 30 patients).

Several ATN profiles were uncommon in our patient population, overall and in each diagnosis group. A−T+N−, A−T+N+, and A+T−N+ were each found in approximately ≤5% of the patients when data-driven cutoffs were used (Table 1). A+T+N+ was uncommon among the SCI and MCI groups, but most common in the dementia group.

### Demographics, Cognition, and Clinical Characteristics Across the ATN Profiles

Patient characteristics and comparison of the 3 main ATN categories are summarized in Table 2 (individual ATN profiles in eTable 2, Supplement, links.lww.com/WNL/C301). Characteristics are listed for the data-driven classification (Aβ42 as A).

**Table 2:**
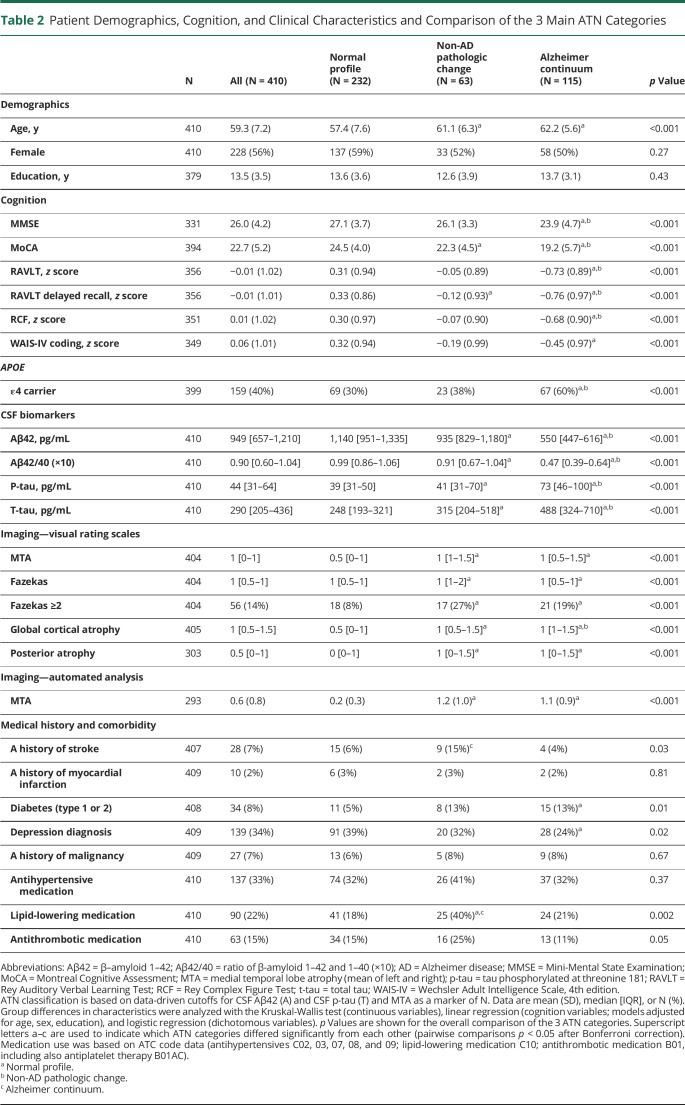
Patient Demographics, Cognition, and Clinical Characteristics and Comparison of the 3 Main ATN Categories

Age increased with increasing biomarker positivity (*p* < 0.001, Table 2), and all A+ groups and A−T+N− were older than A−T−N− (eTable 2, links.lww.com/WNL/C301). There were no differences in sex or education. Patients in the Alzheimer continuum were more often *APOE* ε4 carriers (60%) than patients with non-AD pathologic change (38%) and normal profile (30%) (*p* < 0.001, eTable 2, links.lww.com/WNL/C301).

Cognition worsened with increasing biomarker positivity, with Alzheimer continuum and, particularly, A+T+N+ having the poorest performance (*p* < 0.001 for overall comparisons, Table 2 and eTable 2, links.lww.com/WNL/C301). In the 8-profile comparison, A+ profiles A+T+N−, A+T−N+, and A+T+N+, but not A+T−N−, showed poorer global cognition than A−T−N−. All A+ profiles had poorer memory than A−T−N−. Some differences were also observed between A+ profiles and A−T+N− and A−T−N+. In processing speed/attention, A+T−N+ and A+T+N+, but not A+T−N− or A+T+N−, performed worse than A−T−N−.

Group differences in CSF and MTA were as expected (Table 2 and eTable 2, links.lww.com/WNL/C301). T-tau (marker of N; not used in our ATN classification) was higher in all T+ groups, reflecting the strong p-tau/t-tau correlation. Group differences were also found in white matter hyperintensity, GCA, and PA visual ratings. Compared with A−T−N−, both Alzheimer continuum and non-AD pathologic change had higher Fazekas scores; vascular pathology and comorbidity seemed most pronounced in the latter group (Table 2 and eTable 2, links.lww.com/WNL/C301). Depression was a common comorbidity (34% of all patients, Table 2), with the highest prevalence in the normal group (39%) and lowest in Alzheimer continuum (24%).

### Eligibility for Anti-Aβ Treatment

Of the 404 patients with available data to assess eligibility, clinical diagnosis disqualified more than a half (SCI N = 212, non-AD dementia or MCI N = 35); 43 patients had too low MMSE/MoCA. MRI was missing for 10 patients; 1 patient was excluded because of anticoagulant use. Remaining patients were assessed for biomarker status. Table 3 summarizes what proportion of patients met the criteria with different biomarker cutoffs and definitions of AD-consistent biomarker profile.

**Table 3:**
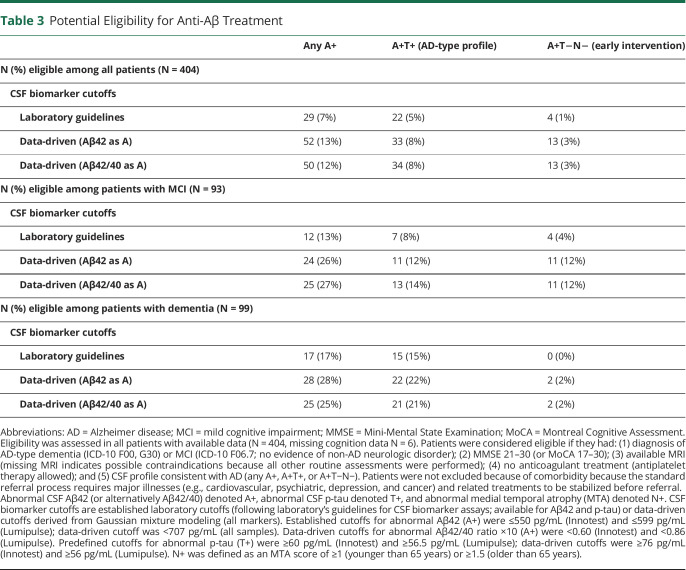
Potential Eligibility for Anti-Aβ Treatment

Overall, when laboratory cutoffs were used for biomarker positivity and any A+ was considered sufficient evidence for AD pathology, 7% of all patients met the anti-Aβ treatment eligibility criteria (Table 3). In the MCI and dementia groups, 13% and 17% met the criteria, respectively. Defining the AD CSF profile as A+T+ (or A+T−N−) led to 5% (1%) of all patients, 8% (4%) of patients with MCI, and 15% (0%) of patients with dementia meeting the eligibility criteria.

Using data-driven biomarker cutoffs increased the number of eligible patients; there was no major difference between the 2 A markers. In total, 12%–13% of the whole cohort (26%–27% of patients with MCI and 25%–28% of patients with dementia) met the criteria when any A+ profile was considered sufficient evidence for pathology. Defining the AD profile as A+T+ or A+T−N− reduced the number of eligible patients, but the pattern was different in the diagnostic groups. Requirement of A+T+ excluded approximately half of the patients with MCI who were eligible based on A+ (decrease from 26%–27% to 12%–14%), while the proportion of eligible patients remained fairly stable in the dementia group (decrease from 25%–28% to 21%–22%). Requirement of A+T−N− excluded all except for 2 patients with dementia but did not affect the MCI group.

## Discussion

In this study, we applied ATN to a large patient group from a Swedish university hospital memory clinic where CSF and neuroimaging are part of the standard diagnostic evaluation. This represents a real-world “best-case scenario” where the necessary specialized diagnostic infrastructure and national guidelines for dementia diagnostic examination are already in place. Regardless of classification cutoffs, most patients had the normal A−T−N− profile (54%–57%) and A+ prevalence was lower than previously reported (17%–30%). Simulation of anti-Aβ treatment eligibility following the published recommendations^21^ (but without considering all safety aspects) showed that up to 13% of the whole cohort (27% of those with MCI and 28% of those with dementia diagnosis) met the eligibility criteria when any A+ profile was considered sufficient biomarker evidence. Defining the AD CSF profile as A+T+ targeted the treatment more to the dementia than MCI stage (eligibility up to 14% in MCI and 22% in dementia). The opposite applied to earlier-stage intervention (A+T−N−; eligibility up to 12% in MCI and 2% in dementia).

Therapeutic decision-making in clinical practice is an important potential future use for ATN. We assessed eligibility for anti-Aβ treatment in a memory clinic with all highly specialized resources required for such treatments. These types of studies are needed to inform the ongoing discussion around the future clinical implementation of disease-modifying treatments. We are not aware of any previous studies in similar settings, but a recent Medicare study tested the aducanumab trial eligibility criteria (without biomarker and cognitive assessments).^41^ More than 90% of patients with AD dementia and 85% of patients with MCI were estimated to meet at least one of the exclusion criteria (usually old age and comorbidity). In our analysis, comorbidity did not affect the eligibility estimates because unstable medical conditions contraindicating treatment need to be stabilized before the memory clinic examination, as per the standard referral process. Owing to incomplete comorbidity data, it is nevertheless possible that our analysis overestimates eligibility. In general, comorbidity contraindicating treatment may be more common at older ages than in the somewhat younger patients referred to our highly specialized memory clinic.^42,43^

Whether ATN is a suitable approach for diagnostic and therapeutic decision-making in clinical practice is currently unclear. Testing in real-life clinic settings and assessing the effect of different operationalization choices (definition and number of biomarker profiles, choice of biomarkers, and cutoffs) is required to address this question. ATN has so far been explored primarily in selected research cohorts, with variation in profile prevalence. In our study, A−T−N− was more common and A+ profiles less common than in research cohorts. Depending on cutoffs and A marker, the prevalence of A−T−N− was in our study 76%–82% in those with SCI, 38%–46% in those with MCI, and 14%–17% in those with dementia. Previous studies reported a prevalence of 32%–61% in SCI/cognitively healthy individuals,^6,7,9-11,13,14,17,20^ 8%–41% in those with MCI,^9-11,16,20^ and 3%–11% in those with AD-type dementia.^8,10,11,16^ The prevalence of A+ (A+T+) was in our study 3%–13% (1%–5%) in those with SCI, 18%–33% (10%–18%) in those with MCI, and 45%–71% (35%–51%) in those with dementia, whereas in previous studies, it was 18%–40% (5%–21%) in SCI/cognitively healthy individuals,^6,7,9-11,13,14,17,20^ 42%–91% (18%–84%) in those with MCI,^9-11,16,20^ and 70%–92% (42%–83%) in those with AD-type dementia.^8,10,11,16^ Using an ATN operationalization similar to ours (data-driven cutoffs for CSF Aβ42 and p-tau as A and T, imaging as N), the Swedish BioFINDER study (including 2 cohorts) reported an A−T−N− prevalence of 37% among cognitively unimpaired participants (both cohorts) and 2% among patients with MCI/AD dementia (cohort 2: 22%).^40^ A+ prevalence was 49% and 96% in these groups (cohort 2: 35% and 58%); A+T+ prevalence was 17% and 85% (cohort 2: 16% and 39%).^40^

Few studies have so far been conducted in real-life memory clinics and unselected populations. In the ABIDE project (Amsterdam memory clinic), A−T−N− was less common (SCI 48%, MCI 19%, and any dementia 6%) and A+ more common (SCI 21%, MCI 51%, and any dementia 66%) than in our clinic.^15^ A+T+ prevalence (SCI 2%, MCI 16%, and any dementia 42%) fell within the range observed in our study.^15^ Of importance, ABIDE used amyloid PET as A marker, i.e., the difference in A+ prevalence may be even larger. Amyloid PET becomes abnormal later than CSF Aβ,^44^ and replacing amyloid PET with CSF Aβ as A marker increases the A+ prevalence.^40^

Several ATN profiles were uncommon among our patients, including A−T+N−, A−T+N+, and A+T−N+ (overall and in each diagnostic group) and A+T−N− and A+T+N+ in certain groups (A+T−N− in the SCI and dementia groups, A+T+N+ in the SCI and MCI groups). These profiles were also underrepresented in many other cohorts,^6,10,15,17,40^ making it difficult to meaningfully characterize between-profile differences. This is an important consideration when potentially expanding the system toward ATXN by adding biomarkers for other pathologies^44^ and increasing the number of profiles. Regarding the more common ATN profiles, our findings were similar to previous reports, e.g., older age, a higher percentage of *APOE* ε4 carriers, and poorer cognition among A+T+ vs A−T−N−,^9,15,20^ and a higher prevalence of vascular pathology and comorbidity in non-AD pathologic change (alone or concomitant with AD).^15^

Another key consideration in ATN is the choice of A, T, and N markers because internal concordance between different markers (e.g., fluid vs imaging) remains to be fully established. CSF Aβ and amyloid PET are both considered valid markers of A,^2,44^ and they are equally acknowledged in the recommendations for anti-Aβ treatment.^21^ Regarding the different CSF Aβ markers, Aβ42/40 could be preferred over Aβ42 because it corrects for preanalytical confounding and individual differences in Aβ production and correlates better with PET and disease progression.^45,46^ We found that replacing Aβ42 with Aβ42/40 as A resulted in a decrease in A+T−N+ but increase in A+T+, pointing to a stronger correlation between Aβ42/40 and p-tau (T) and suggesting potential non-AD pathology among patients with discrepant A markers. In line with this, a French memory clinic study reported mostly normal Aβ42/40 levels in A+T− patients and abnormal levels in A−T+ patients.^47^ These results support using Aβ42/40 in clinical practice as a CSF marker of A. Aβ42/40 could be particularly informative when Aβ42 and other markers are discrepant.^46,48^

Because widely accepted biomarker cutoffs do not yet exist, the normal/abnormal dichotomy in the ATN system is a challenge. A common and pragmatic approach is to use established laboratory cutoffs for CSF biomarkers and visual rating of brain atrophy or amyloid PET,^15,16,20^ but data-driven, e.g., GMM-based cutoffs, have become an attractive alternative.^9,40^ Particularly for CSF Aβ42, laboratory cutoffs have been suggested to underestimate abnormality, and studies have reported higher Aβ42 cutoffs with data-driven methods^37,38^ (tested also with CSF tau^39^). In line with previous studies, our data-driven cutoff for CSF Aβ42 (707 pg/mL) was higher than the laboratory cutoff, and it fell within the range of previously reported GMM cutoffs for similar Aβ42 assays (680–813 pg/mL^37,38,40^). P-tau cutoffs (76 and 56 pg/mL) were slightly higher than those previously reported (50.5–56 pg/mL^39,40^). Our key finding was that data-driven cutoffs led to an increase in A+ (and A+T+) prevalence and decrease in the prevalence of non-AD pathologic change. However, A+ prevalence was lower than that previously reported, and several ATN profiles were uncommon, regardless of cutoffs. While data-driven methods are convenient tools for research, it should be noted that data-driven cutoffs are study-specific and dependent on sample characteristics. The clinical applicability and relevance of different cutoffs and appropriate methods to assess biomarker status and eligibility for disease-modifying treatments in clinical practice remain open questions.^49^

We applied the ATN system and evaluated anti-Aβ treatment eligibility in a large, well-characterized, and heterogeneous real-life memory clinic cohort. This is a typical example of a clinic with all the highly specialized resources required for both diagnostic assessments and implementation of anti-Aβ and/or other disease-modifying treatments. Irrespective of the reason for referral, most patients undergo comprehensive CSF and imaging assessments routinely. In our study, patients who did vs did not undergo LP were similar regarding demographics and cognition. Another strength is that we explored both laboratory and data-driven biomarker cutoffs, compared CSF Aβ42 and Aβ42/40 as A markers, and used MTA as a clinically pragmatic, well-established N marker. Given the strong CSF p-tau/t-tau correlation, using different ATN biomarker measurement modalities (fluid and imaging) is recommended.^9^

Our population was somewhat younger than those in previous similar studies, e.g. ABIDE (average difference approximately 3–6 years depending on diagnosis^15^). Distribution of diagnostic groups was also different, i.e., half of our patients experienced SCI and a quarter had dementia, whereas one-third of the ABIDE patients experienced SCI and half had dementia.^15^ This may be at least partly due to different referral systems, e.g., earlier referrals to the Karolinska clinic, which also has responsibility for individuals younger than 70 years in the entire Stockholm region. In addition, in Sweden, many dementia cases with standard presentations are diagnosed and managed in primary care. Our findings may thus not be representative of other memory clinics or of the general population with dementia-related diseases. Given that Aβ/biomarker abnormalities increase with increasing age and degree of cognitive impairment,^6,50^ the age and diagnosis distribution might at least partly explain the observed higher A−T−N− prevalence and lower A+ prevalence. Our study is thus most representative of highly specialized clinical settings where new disease-modifying treatments could be initiated in earlier disease stages when they may have greater chance of clinical benefit and more favorable risk-benefit ratio. Regarding anti-Aβ treatment eligibility, we could not assess all safety-related exclusion criteria including those potentially listed in full MRI reports. Our estimates might thus overestimate actual eligibility.

Applying the ATN biomarker system to a real-life memory clinic cohort showed that its implementation into clinical practice is challenging. Important issues such as biomarker cutoffs and optimal number of pathology profiles remain to be resolved. The fact that most patients had normal Aβ and were deemed ineligible for anti-Aβ treatment underlines the complexity of cognitive disorders and the need for disease-modifying therapies with other mechanisms of action.

## Glossary

Aβ: β-amyloid
AD: Alzheimer disease
ATN: β-amyloid, Tau, Neurodegeneration biomarker classification system
DSM: Diagnostic and Statistical Manual of Mental Disorders
GCA: global cortical atrophy
GMM: Gaussian mixture modelling
LP: lumbar puncture
MCI: mild cognitive impairment
MMSE: Mini-Mental State Examination
MoCA: Montreal Cognitive Assessment
MTA: medial temporal lobe atrophy
PA: posterior atrophy
p-tau: phosphorylated tau 181
SCI: subjective cognitive impairment
t-tau: total tau
WMH: white matter hyperintensity
